# Titania as Buffer Layer for Cd-Free Kesterite Solar Cells

**DOI:** 10.1021/acsmaterialslett.2c00933

**Published:** 2022-12-19

**Authors:** Giorgio Tseberlidis, Valerio Di Palma, Vanira Trifiletti, Luigi Frioni, Matteo Valentini, Claudia Malerba, Alberto Mittiga, Maurizio Acciarri, Simona O. Binetti

**Affiliations:** † Department of Materials Science and Solar Energy Research Center (MIB-SOLAR), University of Milano-Bicocca, Via Cozzi 55, 20125, Milano, Italy; ‡ ENEA C.R. CASACCIA, Via Anguillarese 301, 00123, Roma, Italy

## Abstract

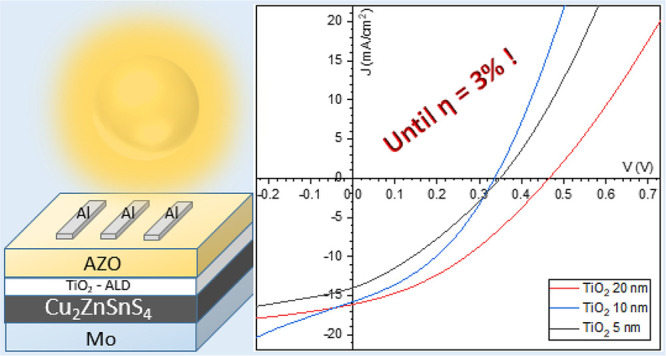

Pure sulfide kesterite (Cu_2_ZnSnS_4_) is one of the most promising emerging photovoltaic technologies thanks to its excellent absorption coefficient, cost-effectiveness, and environmental sustainability. However, record efficiencies are not exceeding 11% due to several issues, such as absorber defects or a nonoptimal band alignment with the toxic but conventionally used CdS buffer layer. To get rid of it, several efforts have been made in the past few years. Among recent theoretical works, TiO_2_ has been suggested as a suitable buffer layer due to its optical and electrical properties, giving extremely promising results in device simulation. However, there are few experimental examples combining TiO_2_ with kesterite, and they generally show very modest performances. In this Letter, we report on the preliminary and promising results of our experimental procedure for the production of Cd-free kesterite photovoltaic devices featuring ALD-TiO_2_ as a buffer layer, leading to efficiencies comparable with our CZTS/CdS reference devices.

Kesterite occurs in nature as a mineral with the formula Cu_2_(Zn,Fe)SnS_4_ where zinc and iron share the same position in the crystalline unit.^1^ In the last 20 years, this material attracted the attention of the photovoltaic (PV) community as a p-type semiconductor, thanks to its excellent absorption coefficient, the earth-abundance of its components, and thus, its potential application in low-cost thin-film solar devices.^2^ In 2012, the 12.6% record efficiency for this class of materials was reached for the low bandgap (1.1 eV) selenized kesterite (CZTSSe),^3^ while in 2018, the record efficiency of 11% was recorded by Yan and co-workers for the pure sulfide, wide bandgap (∼1.5 eV), kesterite Cu_2_ZnSnS_4_ (CZTS) always adopting CdS as a buffer layer.^4^ CZTS is one of the most promising absorbers among the emerging photovoltaic technologies, and thanks to its bandgap of around 1.5 eV, it can be a suitable candidate for tandem device architectures.^5^ Despite its apparent compositional similarity with CuInGaSe_2_ (CIGS), kesterite solar cells never reached efficiencies comparable to CIGS thin films, due to a *V*_oc_ deficit mainly caused by point defects and a nonoptimal band alignment with CdS, which is commonly used as the n-type partner.^6^ Several efforts have been made to get rid of a toxic and nonindustrially appealing buffer layer such as CdS.^7^ Among the cheapest nontoxic alternatives, ZnSnO and Zn(O,S) are the most promising. Specifically, the CZTS/ZnSnO junction exhibited power conversion efficiencies comparable to those of CZTS/CdS junction.^7^ Among the other possibly suitable n-type semiconductors, recent theoretical models indicate TiO_2_ as one of the best candidates to be coupled with CZTS.^8−11^ TiO_2_ has been widely employed in dye-sensitized solar cells (DSSC) and perovskite solar cells because of its transparency, transport properties and low-cost precursors,^12^ but very little experimental evidence has been recorded of TiO_2_ coupled with CIGS or CZTS in a p–n junction.^13−19^ Calculations of Bencherif and co-workers indicate that a CZTS/TiO_2_ junction could lead to a ∼ 5% *V*_oc_ gain compared to canonical CZTS/CdS, while the simulated optimized solar cell should be able to overcome also the experimental record Fill Factor (FF) values leading to the overall power efficiency of ∼15%.^11^ On the other hand, Nisika and co-workers widely studied the properties of the CZTS/amorphous-TiO_2_ where a favorable spike-like effect at the p–n junction interface leads to an offset conduction band value of 0.17 eV. A few years later, the same authors reported a study where the influence of TiO_2_ oxygen vacancies on charge transfer mobility has been systematically investigated, revealing that a lower concentration of oxygen is essential for an efficient charge extraction.^9,18^ Despite the encouraging simulations and calculations, only a few experimental works have been reported on this topic. Demopoulos and co-workers have been pioneers when in 2015 developed a sophisticated but effective synthetic route to grow CZTS nanocrystallites on TiO_2_ nanorod arrays (TiO_2_-NA) and used them to produce the first working solar cell (in superstrate configuration, TCO/TiO_2_/interlayer/CZTS-NA/top-contact) showing extremely modest parameters, such as *V*_oc_ ∼ 180 mV and *J*_sc_ ∼ 3.3 mA/cm^2^.^13^ In 2019, again Demopoulos et al., optimized their TiO_2_–NA synthetic procedure reaching efficiencies ∼1% with *V*_oc_ ∼ 400 mV and *J*_sc_ ∼ 6.6 mA/cm^2^, always in a sort of superstrate configuration and with an Al_2_O_3_ interlayer between CZTS and TiO_2_–NA, converting the architecture to a *p-i-n* junction.^17^ More recently also Wang and co-workers reported an Ag-substituted Se-based kesterite solar cell but always in superstrate configuration, featuring a sputtered layer of TiO_2_ with extremely interesting results.^20^ In the meantime, other works have been reported employing TiO_2_ in CZTS solar cells but interposing a thin layer of CdS between them, thus slightly modifying the band alignment and resulting in efficiencies <1%.^14,16^ An interesting report has been recently published by Dwivedi et al., with a superstrate solar cell configuration featuring commercial TiO_2_ and CZTS both deposited via straightforward wet chemistry procedure but reaching only η = 0.87%, *V*_oc_ = 400 mV, and *J*_sc_ = 4.7 mA/cm^2^ as device parameters.^15^ In this work, we report the preliminary results of our experimental procedure for the production of Cd-free fully sulfurized kesterite PV devices (in conventional substrate configuration) featuring ALD-deposited TiO_2_ as a buffer layer. To the best of our knowledge, this is the current record device for CZTS/TiO_2_ heterojunction in substrate configuration with a power conversion efficiency of 3.01% with a respectable *J*_sc_ of ∼16 mA/cm^2^ and *V*_oc_ ∼ 460 mV.

In the first instance, the transmittance of the ALD-TiO_2_ layers has been tracked through UV–vis measurements on samples grown on bare SLG. In Figure 1a, it is possible to notice a higher transmittance of TiO_2_, in the region between 300 and 500 nm, compared to CdS (deposited also on SLG by chemical bath deposition, as described elsewhere^21,22^) thus confirming that its substitution with TiO_2_ could allow a better solar light harvesting by minimizing the parasitic absorptions in that region. Moreover, the band gap calculated from Tauc’s Plot on TiO_2_ and CdS absorbance spectra (Figure 1b and 1c) gives values, respectively, of 3.2 and 2.4 eV, in accordance with others cited in literature.^23,24^

**Figure 1:**
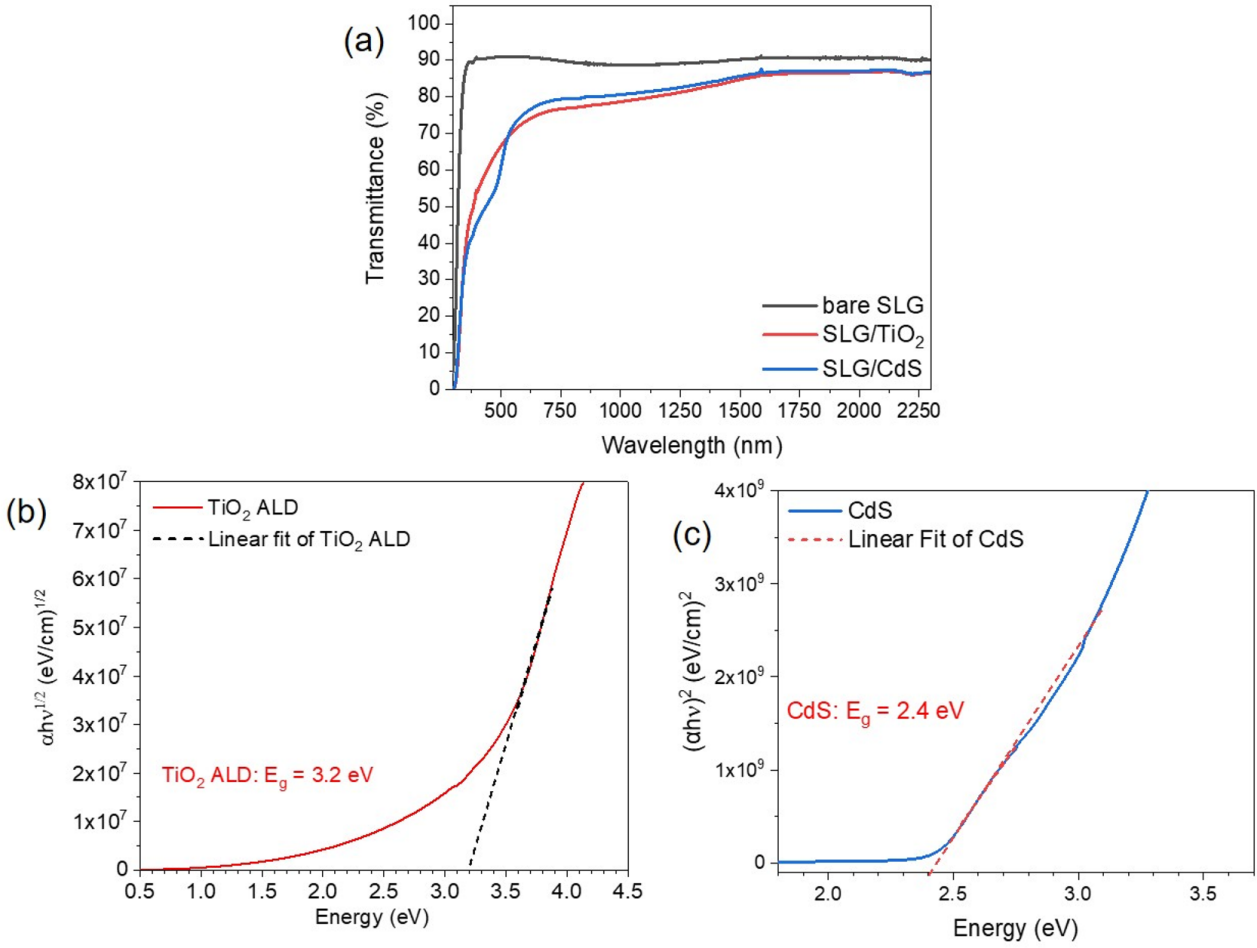
(a) Transmittance spectra of bare SLG, SLG/TiO_2_, and SLG/CdS; (b) Tauc plot and calculated band gap for ALD-TiO_2_; (c) Tauc plot and calculated band gap for CdS.

First, to have a yardstick, our typical CZTS solar cell with standard architecture Mo/CZTS/CdS/i-ZnO/AZO/Al has been produced and measured. In Table 1 (entry 1) are reported the average values of the reference CZTS/CdS cell parameters: an average efficiency η = 4.14% has been obtained with *V*_oc_ = 555 mV, *J*_sc_ = 12.5 mA/cm^2^, FF = 60%. Then, to highlight the buffer layer role in the p–n junction, samples with architectures Mo/CZTS/i-ZnO/AZO/Al and Mo/CZTS/AZO/Al have been produced and measured, giving no diode-like behavior, as expected. Subsequently, by using our standard cell architecture and substituting only CdS with TiO_2_ (Mo/CZTS/TiO_2_/i-ZnO/AZO/Al), no working solar device was obtained, regardless of the TiO_2_ thickness used. Given the already known possible high resistivity of TiO_2_,^25−27^ the second samples data set has been produced with the same methodology, but without i-ZnO interlayer, thus completing the device just with AZO and Al grid (Mo/CZTS/TiO_2_/AZO/Al). The parameters of the new working devices are reported in Table 1 (entries 2–4), where it is possible to notice better performances for TiO_2_ thickness of 20 nm (Figure 2). In particular, a 2.77% of efficiency has been obtained for the champion device, which reached a power conversion efficiency of 3.01% after 1 h of light soaking, thus suggesting that these solar cells could be even more efficient when used in normal operating conditions.^28,29^ This is, to the best of our knowledge, the highest efficiency ever reported for a CZTS/TiO_2_ p–n junction, and in addition to this, it is close and comparable to our CZTS/CdS reference device which shows an average η = 4.14%. In Table 1, the single diode fitting parameters are also reported. In the case of the device with 20 nm of TiO_2_ an ideality factor of *n* = 1.95, similar to our CZTS/CdS reference device, is obtained, while worse values for *J*_0_, *R*_s_, and *R*_sh_ are found. After the light soaking, the *R*_s_ and *R*_sh_ values are slightly improved and are responsible for the FF% and η% increase. However, by decreasing the TiO_2_ thickness, the fitting model becomes less reliable with *n* > 2, the typical behavior of devices with strong interface recombination.^22,30,31^ In fact, also *R*_s_ and *R*_sh_ show worse values compared to the CZTS/CdS reference device, strongly affecting the FF% and so the final device performance. This feature may be related to a still nonoptimal interface between the CZTS absorber and the new TiO_2_ buffer layer, which can be however improved in future by properly tuning the oxygen vacancies thanks to the optimization of the ALD parameters and conditions.

**Table 1:** Device Parameters of the Champion Devices

entry	cell architecture	buffer layer	*V*_oc_ (mV)	*J*_sc_ (mA/cm^2^)	FF (%)	η (%)	*R*_sh_ (Ω cm^2^)	*R*_s_ (Ω cm^2^)	*J*_0_ (A/cm^2^)	*n*
1	SLG/Mo/CZTS/CdS/i-ZnO/AZO/Al	CdS	555	12.5	59.7	4.14	400.0	0.54	2.20 × 10^–07^	1.99
2	SLG/Mo/CZTS/TiO_2_/AZO/Al	TiO_2_ 20 nm	466	16.1	37.0	2.77	95.2	11.32	1.50 × 10^–06^	1.95
			465^a^	16.5^a^	39.1^a^	3.01^a^	110.8^a^	10.40^a^	1.50 × 10^–06^^a^	1.95^a^
3	SLG/Mo/CZTS/TiO_2_/AZO/Al	TiO_2_ 10 nm	334	15.8	37.0	1.95	62.6	7.10	3.10 × 10^–05^	2.21
4	SLG/Mo/CZTS/TiO_2_/AZO/Al	TiO_2_ 5 nm	347	13.8	31.0	1.48	59.9	6.98	2.10 × 10^–04^	3.59

a After 1 h of light soaking.

**Figure 2:**
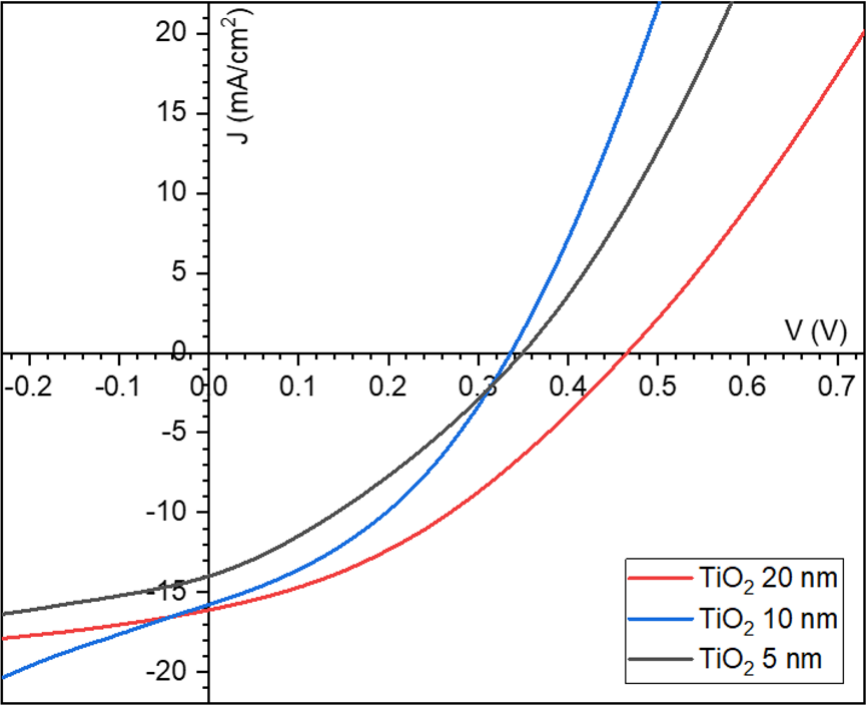
*J–V* curves for the champion devices with CZTS/TiO_2_ junction and three different thicknesses of TiO_2_ buffer layer.

Figure 3 reports the box plots of the cell parameters, where it is possible to notice a narrow range of values for all the measured devices, thus attesting to the good quality and homogeneity of all the samples.

**Figure 3:**
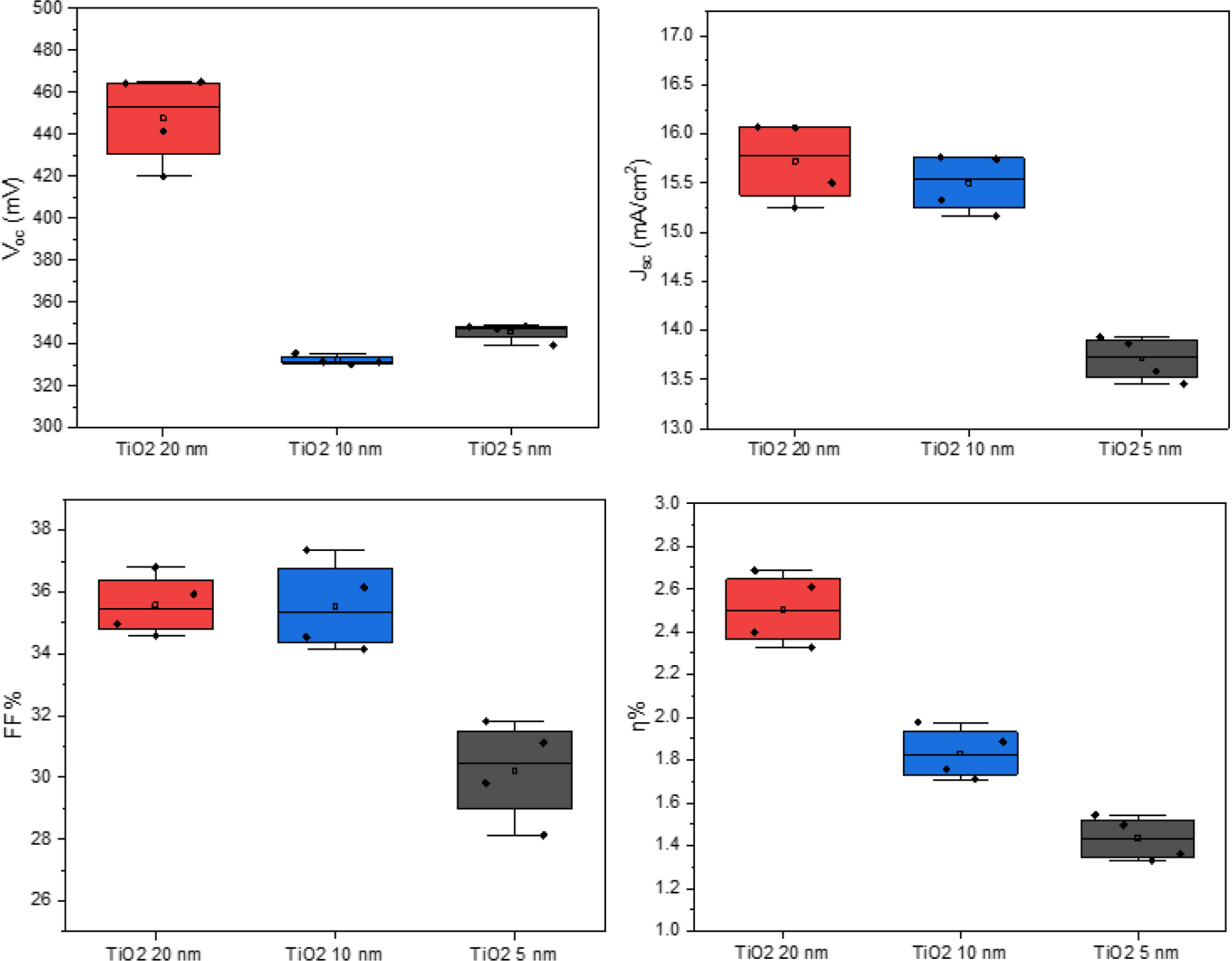
Box plot of the average values for the devices with CZTS/TiO_2_ junction and three different thicknesses of TiO_2_ buffer layer.

From EQE measurements (Figure 4), it is possible again to notice a better performance for the device featuring 20 nm of TiO_2_ as the buffer layer. Moreover, by comparison with CZTS/CdS reference device, it is evident a gain in efficiency in the region around 300–500 nm where CdS shows its peak of absorbance. The current density calculated from the EQE curves is slightly lower compared to the ones obtained from JV measurements, due to the partial shading of the light spot from the Al grid: it results in 13.4 mA/cm^2^ for the device with 20 nm TiO_2_ and ∼12 mA/cm^2^ for the other two devices with lower titania thickness and the reference device with the CdS buffer layer. Despite this, the overall quantum efficiency of the devices ranges between 50% and 65% in the visible region proving the good solar cells’ performance, and again, it is comparable to the 65% EQE of the CZTS/CdS reference device. In addition to this, the curves are good-shaped, especially in the region between 600 and 700 nm, with no abrupt slope, which may suggest the reduction of the recombination compared to the CZTS/CdS reference device.

**Figure 4:**
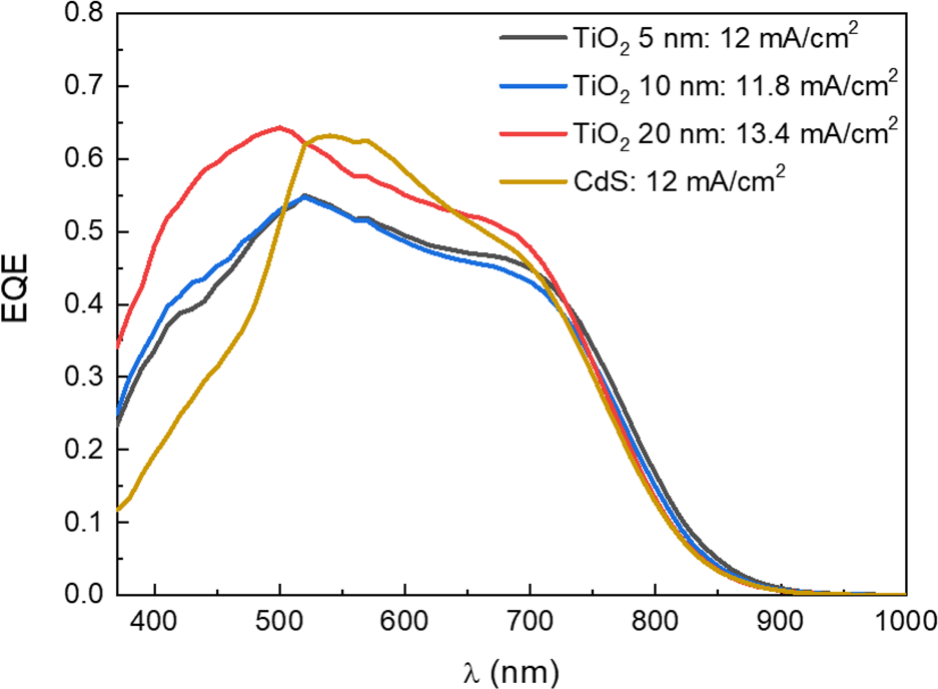
EQE measurements for the reference device with CZTS/CdS junction and the devices with CZTS/TiO_2_ junction with three different thicknesses of TiO_2_ and their current density calculated from EQE, respectively: 12 mA/cm^2^ (CdS), 13.4 mA/cm^2^ (TiO_2_ 20 nm), 11.8 mA/cm^2^ (TiO_2_ 10 nm), and 12 mA/cm^2^ (TiO_2_ 5 nm).

In conclusion, in this Letter, we report our novel and promising procedure for the production of efficient Cd-free kesterite solar cells featuring 20 nm of TiO_2_ as a buffer layer deposited by O_2_-plasma-ALD. We experimentally demonstrate that TiO_2_ can be a good candidate as an n-type partner for CZTS in order to get rid of the toxic and, with conduction bands nonoptimally aligned, CdS.

Compared to the few other experimental works reported on this topic, featuring extremely low performances, we established a new record efficiency of η = 3.01% with the simplest possible cell architecture and with no need for interlayers between CZTS and TiO_2_. Moreover, the results obtained with CZTS/TiO_2_ devices are comparable to the ones with our ∼4% efficiency CZTS/CdS reference cells, suggesting that, by applying our ALD-TiO_2_ deposition procedure on a higher quality CZTS, much higher performances could be obtained. The light-soaking experiment allows predicting that the cells can work even better in normal operating conditions and, supported by literature, we suppose that this could be related to an increase of oxygen vacancies in the titania thin-film composition.^25−27^ Further investigations on TiO_2_ oxygen vacancies will be carried out, for example by exploring thermal-ALD or postdeposition treatments on the device in order to meet the theoretical calculated performances and improve the device behavior, especially in terms of series and shunt resistance aiming to fully overcome CZTS/CdS-based devices.

## Experimental Methods

### Substrate Preparation

Commercial soda-lime glass (SLG) was cleaned in an ultrasonic bath with the following procedure: mucasol solution (15′), deionized water (3 × 15′), acetone (15′), and ethanol (15′). Then the substrates were dried one by one in vacuum and finally coated with a Mo thin film deposited by magnetron DC sputtering in two steps, with a final thickness of 1.1 μm.

### CZTS Thin Film Deposition

CZTS absorber was grown by a two-step process as already described elsewhere:^32^ (i) cosputtering deposition of a 900 nm thick quaternary precursor layer from three targets of Cu, ZnS and SnS at a working pressure of 5 × 10^–3^ mbar and (ii) thermal treatment at 580 °C for 1 h in sulfur atmosphere (sulfurization), necessary to introduce the correct amount of sulfur into the absorber and to promote the grain growth. The sputtering powers applied to each target were properly settled in order to confine the final metal ratios close to the optimum range for photovoltaic application (Cu/Sn ratio within 1.7–1.8 and Zn/Sn ∼ 1.2).^33,34^

### TiO_2_ Plasma-ALD Deposition

TiO_2_ thin layers have been deposited by O_2_-plasma-ALD with a substrate temperature of 250 °C, by using a PICOSUN R-200 Advanced ALD system, equipped with a remote inductively coupled plasma source, operating at 3.2 MHz (Ar = 40 sccm and O_2_ = 190 sccm, with N_2_ as gas carrier at 150 sccm). The precursor tetrakis(dimethylamido)titanium(IV) [(Me_2_N)_4_Ti, TDMATi, purchased by Strem Chemicals] has been maintained at 67 °C throughout the deposition with its respective gas-line at 75 °C to avoid precursor condensation. N_2_ flowing at 150 sccm was used as carrier gas and for the line purge. The cycle was composed of a sequence of Ti:N_2_:O_2_-plasma:N_2_ with pulse duration respectively of 0.6:15:17:4s. The described process showed a nominal growth rate of 0.67 Å/cycle, allowing the deposition of three different desired thicknesses (20, 10, and 5 nm).

### TiO_2_ Characterization

TiO_2_ thickness was determined by spectroscopic ellipsometry with a Film Sense FS-1 ellipsometer system, using a Tauc–Lorentz oscillator for the fitting of the optical constants. The measurement was performed ex-situ onto a piece of c-Si placed together with the SLG/Mo/CZTS substrates during the deposition.

### Device Preparation and Measurements

All the CZTS/TiO_2_ samples have been finalized to solar cells by deposition of RF-sputtered i-ZnO (70 nm) and Al-doped ZnO (AZO) chosen as top contact and deposited by DC pulsed (2 kHz) sputtering with a thickness of 350 nm. In other cases (where specified), the CZTS/TiO_2_ samples have been finalized to solar cells directly with the AZO sputtered layer without the i-ZnO interlayer. Finally, the devices were completed by evaporation of an Al grid (thickness ∼500 nm). The PV cells were scribed manually into isolated areas of 0.11 cm^2^ and have been characterized using a 500 W xenon light source (ABET Technologies Sun 2000 class ABA Solar Simulator), calibrated to AM 1.5 (100 mW/cm^2^) by a reference Si cell photodiode and an IR cutoff filter (KG-5, Schott) to reduce the mismatch between the simulated light and the AM 1.5 spectrum in the 350–750 nm range. The IV curves were measured by applying an external bias to the device, and recording the generated photocurrent with a Keithley model 2400 digital source meter. External quantum efficiency (EQE) measurements were recorded using a custom system based on a monochromator (SPEX MINIMATE 1681 B) with single grating in Czerny–Turner optical design and on a lock-in amplifier working at a chopping frequency of 32 Hz.
